# Analysis of IoT Security Challenges and Its Solutions Using Artificial Intelligence

**DOI:** 10.3390/brainsci13040683

**Published:** 2023-04-19

**Authors:** Tehseen Mazhar, Dhani Bux Talpur, Tamara Al Shloul, Yazeed Yasin Ghadi, Inayatul Haq, Inam Ullah, Khmaies Ouahada, Habib Hamam

**Affiliations:** 1 Department of Computer Science, Virtual University, Lahore 55150, Pakistan; 2 Department of Information and Computing, University of Sufism and Modern Sciences, Bhit Shah 70140, Pakistan; 3 Department of General Education, Liwa College of Technology, Abu Dhabi 15222, United Arab Emirates; 4 Department of Computer Science, Al Ain University, Abu Dhabi 112612, United Arab Emirates; 5 School of Information Engineering, Zhengzhou University, Zhengzhou 450001, China; 6 Department of Computer Engineering, Gachon University, Seongnam 13120, Republic of Korea; 7 School of Electrical Engineering, Department of Electrical and Electronic Engineering Science, University of Johannesburg, Johannesburg 2006, South Africa; 8 College of Computer Science and Engineering, University of Ha’il, Ha’il 55476, Saudi Arabia; 9 International Institute of Technology and Management, Commune d’Akanda, Libreville BP 1989, Gabon; 10 Faculty of Engineering, Université de Moncton, Moncton, NB E1A3E9, Canada; 11 Spectrum of Knowledge Production & Skills Development, Sfax 3027, Tunisia

**Keywords:** internet of things, cyberattacks, anomalies, deep learning, machine learning, healthcare

## Abstract

The Internet of Things (IoT) is a well-known technology that has a significant impact on many areas, including connections, work, healthcare, and the economy. IoT has the potential to improve life in a variety of contexts, from smart cities to classrooms, by automating tasks, increasing output, and decreasing anxiety. Cyberattacks and threats, on the other hand, have a significant impact on intelligent IoT applications. Many traditional techniques for protecting the IoT are now ineffective due to new dangers and vulnerabilities. To keep their security procedures, IoT systems of the future will need AI-efficient machine learning and deep learning. The capabilities of artificial intelligence, particularly machine and deep learning solutions, must be used if the next-generation IoT system is to have a continuously changing and up-to-date security system. IoT security intelligence is examined in this paper from every angle available. An innovative method for protecting IoT devices against a variety of cyberattacks is to use machine learning and deep learning to gain information from raw data. Finally, we discuss relevant research issues and potential next steps considering our findings. This article examines how machine learning and deep learning can be used to detect attack patterns in unstructured data and safeguard IoT devices. We discuss the challenges that researchers face, as well as potential future directions for this research area, considering these findings. Anyone with an interest in the IoT or cybersecurity can use this website’s content as a technical resource and reference.

## 1. Introduction

The Internet of Things (IoT) connects everything in the modern world and is gaining traction in business, particularly in healthcare. The IoT is one of the most popular new ideas in recent years. It locates, transmits, and analyzes data using a network of connected components. In the IoT, “things” are sensors, RFID tags, heart rate monitors, and other smart devices that collect and transmit data. New devices are added to IoT networks daily. There will be roughly 20.4 billion connected devices in 2022, up from 8.4 billion in 2020 [1].

The IoT has an impact on our daily social, commercial, and economic activities. IoT revenue is expected to increase from 892 billion USD in 2018 to more than 4 trillion USD by 2025. This expansion is directly related to the growth of the digital economy. The Internet of Things has enabled smart meters, remote monitoring, process automation, smart homes, smart cities, and smart businesses [2]. Current and future Internet of Things applications and services have the potential to significantly improve the ease, speed, and comfort of customers’ lives [3]. Many cyber threats and attacks, however, are significant impediments to IoT development.

Expansion of IoT networks raises significant issues in several areas, including device management, data management, computation, security, and privacy [4]. Several security flaws discovered may jeopardize the burgeoning IoT. Future IoT applications, such as those mentioned above, may fail to fulfill all their promises if a dependable framework is not in place as they will be unable to meet individual needs or adhere to social norms. IoT systems are divided into four layers: the application layer; the middleware or support layer; the networking and data transmission layer; and the perception or sensing layer. There are many layers to IoT applications, and each needs different technology [2]. At each stage, there are particular security concerns and difficulties. Attacks, including denial-of-service, spoofing, jamming, eavesdropping, data manipulation, and man-in-the-middle, are among the most common IoT risks.

Because security threats and attacks are becoming more numerous and complex, traditional security practices are no longer as effective as they once were. Future IoT infrastructure requires a security solution that uses risk-mitigation technology to reduce risk. Proponents of the Fourth Industrial Revolution argue that artificial intelligence (AI) is critical to the future development of intelligent systems. As a result, we can detect unexpected or harmful IoT behaviors and provide a dynamic, adaptive security solution by leveraging artificial intelligence skills, particularly machine, and deep learning. To sift through security data in search of novel insights and trends, machine learning and deep learning models commonly use a preset set of rules, strategies, or complex transfer functions [3]. By recognizing anomalies in the IoT, developed security models might also be used to teach robots how to defend themselves against potential threats or attacks. The paper’s contributions to the body of literature are outlined in the following paragraphs. Data on how these technologies are used in the IoT are being gathered [4]. Numerous academic studies have been conducted on IoT security. For example, some authors have carried out a survey of IoT security vulnerabilities in which they examine and classify common security issues relating to the layered design, networking, communication, and management of the IoT [5]. The findings of a second study on the security of the IoT were published and produced a list of IoT security research opportunities and concerns after taking security in a broad meaning [6]. In addition to discussing IoT simulators and models, it summarizes the current state of IoT security research. The author also gives a quick overview of the principles of IoT security, existing dangers, potential solutions, and projections for this industry’s future [7]. They investigate the problems and state of IoT security in their study at the application, network, and perception layers [8].

The authors consider application domains, security issues, and the process by which solutions are developed. The authors show how attack vectors, vulnerabilities, and other relevant techniques can be used to classify IoT security issues [9]. The authors overview the most recent threats and vulnerabilities related to the IoT by carefully analyzing IoT security research [10]. There have been significant studies on machine learning, in addition to surveys. We look at IoT security solutions based on supervised, unsupervised, and reinforcement learning techniques. Their research primarily focuses on machine-learning-based authentication, access control, safe offloading, and virus detection for IoT data privacy solutions [11]. The authors investigate many concerns, including potential attack vectors and IoT network security requirements. The use of computers and deep learning to secure the IoT is examined [12]. Researchers have analyzed known and unknowable risks, accessible solutions, and barriers to see how the IoT’s increasing capabilities affect security and privacy [13].

Understanding the nature of data, the many kinds of cyber threats, and other pertinent factors is essential when using machine learning and deep learning to build data-driven security systems [14]. Regarding connectivity, the IoT controls how and what happens when things communicate. This suggests that, regardless of location, IoT networks are always available. Networks must remain flexible and responsive because IoT devices are constantly added and removed [15].

Because IoT devices are constantly being added and withdrawn, the network reconfiguration process must be dynamic and flexible. Ad hoc networks may rely on nearby devices for short-range communications [5]. An IoT-enabled device transforms and acts based on its proximity to the current location [16]. Wireless communication is the primary obstacle in industrial IoT networks. Highly reliable, low-latency communication is necessary for sensitive applications, such as traffic monitoring, manufacturing on an assembly line, and medical equipment [17].

An IoT device is a piece of hardware equipped with a sensor that sends data between locations through the internet. Because many sensors are used in a complex system application, the systems should be set up to use fewer resources and cost less [15].

There are different techniques of machine learning and deep learning, such as rule-based techniques, the clustering method, optimization of security features, recurrent neural network, multi-layer perceptron, and classification and regression techniques, used to protect IoT data. Regression and classification techniques are well known and frequently used in IoT machine security. Predicting the outcome of discrete values or categories, such as anomaly, average, or attacks, is a standard definition of classification problems. Clustering algorithms may be very helpful in resolving IoT security issues, such as identifying outliers, anomalies, signatures, fraud, and cyberattacks, by exposing previously hidden patterns and structures in IoT security data. Rule-based systems may be essential to IoT security because they may learn security or policy rules from data. A well-known machine learning technique called association rule learning looks for patterns or relationships between the attributes in a security dataset. This MLP network is used to analyze the NSL-KDD dataset’s malware, explain IoT parameters, detect malicious traffic coming from IoT devices, and create a model for intrusion detection. These enhanced signature properties may simplify the management of large amounts of IoT security data, such as identifying anomalies in IoT network traffic, as part of machine-learning-based security modeling. This article explores how ML and DL can be used to uncover attack patterns from unstructured data and protect IoT devices. We address the difficulties researchers encounter and potential future directions for this study area considering these findings. This study analyzes our current understanding of AI, focusing on the efficacy of machine-learning- and deep-learning-based IoT security solutions. We introduce a variety of machine learning and deep learning architectures and techniques and describe how they can be applied to intelligent security modelling in order to address the problem of IoT security. The abbreviations and their full form are shown in Table 1.

### 1.1. Research Gap

Cyberattacks and threats have a significant impact on intelligent IoT applications. Many traditional techniques for protecting the IoT are now ineffective due to new dangers and vulnerabilities. The capabilities of artificial intelligence, particularly machine and deep learning solutions, must be used if the next-generation IoT system is to have a continuously changing and up-to-date security system. We discussed how machine learning and deep learning can be used to detect attack patterns in unstructured data and safeguard IoT devices. Furthermore, we discuss the challenges that researchers face, as well as potential future directions for this research area.

### 1.2. Structure of Our Article

**Figure 1:**
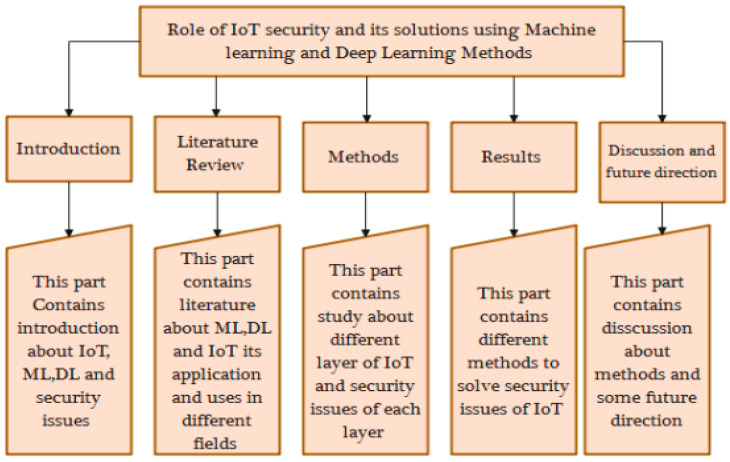
Taxonomy of the study.

The remaining sections of the paper are structured as follows. The background of the domain is covered in Section 2, along with a survey of related works. In Section 3, we explore IoT System Architectures and Security Concerns and our research methodology. Section 4 outlines our research results, potential machine-learning- and deep-learning-based security options for IoT environments. The work is concluded in Section 5. Figure 1 shows the taxonomy of this work.

## 2. Literature Review

The IoT plays an important role in technological advancement. “IoT” stands for “Internet of Things”, and the term “Things” refers to electrical devices that are linked to the internet. The Fourth Industrial Revolution, also known as Industry 4.0, is defined by increased automation of traditional industrial and manufacturing processes. The IoT is one of the intelligent technologies being developed for this movement [18].

The IoT is a network of objects that may connect to the internet and wireless networks to send and receive data automatically. Different organizations and research groups from distinct viewpoints define the IoT and smart environments. The authors claim that RFID-based digital information flows and physical components make up the IoT [19].

The healthcare industry is quickly adopting the IoT, which has the potential to improve patient engagement, health, and access to care. IoT device growth, however, poses significant security, privacy, and safety hazards to patients and healthcare workers. Studies on reducing the risks brought on by the IoT in the healthcare industry are still few and far between. Integrating a secure applications solution with IoT devices in healthcare environments has been the subject of recent research. It is crucial to create a specialized IoT app for health due to the sensitivity of healthcare data and information [20]. Current IoT possibilities for the healthcare sector are promising. They are also quite popular because of their sensing and measuring capabilities, including narrowband IoT in its low-energy variant (N.B. IoT). Because of its low energy consumption, it is favored in the healthcare industry. Several concepts exist for using N.B. IoT in the healthcare industry. It has not been standardized and works flawlessly with cellular systems such as LTE. As a result, N.B. IoT has emerged as a viable option for healthcare-related applications in recent years. However, security measures and other system-related difficulties are the most severe dangers to N.B. IoT. If these concerns and obstacles are addressed appropriately, it has the potential to be one of the most viable and popular solutions for low-power, wide-area healthcare installations [21].

One of the many difficulties facing the Internet of Things which connects a wide range of objects to networks to enable complex and intelligent applications is protecting user privacy and preventing attacks, including spoofing, denial of service (DoS), jamming, and eavesdropping. The author looks into the flaws in IoT systems, as well as possible ways to secure IoT networks using machine learning techniques, such as supervised learning, unsupervised learning, and reinforcement learning (RL). The analysis of data privacy focuses on ML-based approaches for authenticating IoT devices, controlling access to such devices, offloading data securely, and identifying viruses. Future IoT adoption will have a significant effect on society, business, and the economy. Because the majority of nodes in an IoT network have little resources, hackers are drawn to them as easy targets. IoT network security and privacy issues have been addressed in a variety of ways, the majority of which make use of common cryptographic protocols. However, current solutions cannot address the security issues that arise with IoT networks and are exacerbated by the distinctive characteristics of IoT nodes. By implementing machine learning (ML) and deep learning into IoT devices and networks, many threats to the security of the Internet of Things (IoT) can be stopped.

**Figure 2:**
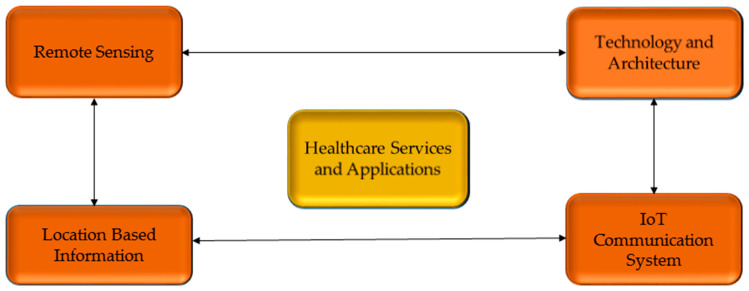
The IoT risk management model in healthcare [23].

Present IoT opportunities in the healthcare sector are promising. It is also well known for its sensing and measuring capabilities, including narrowband IoT in low-energy form (N.B. IoT). It is popular in the healthcare field because of its low energy usage. There are several ideas for using N.B. IoT in the healthcare business. N.B. IoT is already commonplace and works seamlessly with cellular networks such as LTE. As a result, N.B. IoT has emerged as a feasible choice for healthcare-related applications in recent years. The most critical threats to N.B. IoT are security measures and other system-related issues. If these problems and challenges are solved, it has the potential to be one of the most feasible and popular systems for low-power, broad-area healthcare installations [22]. The IoT risk management model in healthcare is presented in Figure 2.

**Figure 3:**
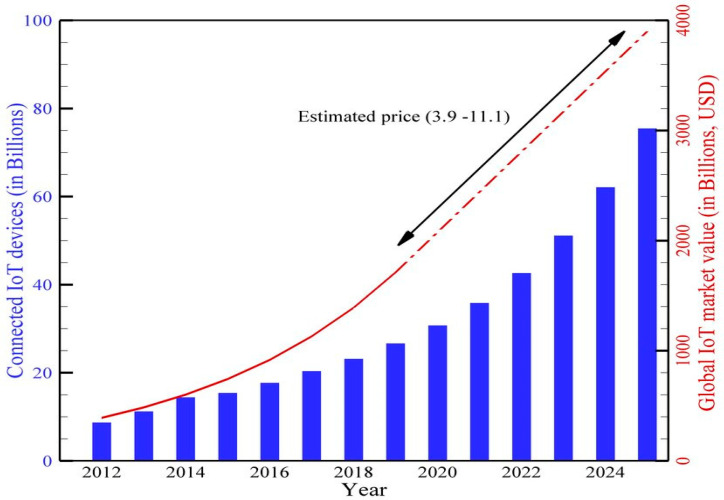
Total connected IoT devices and global IoT market so far and future prediction [25].

The IoT is a way to develop intelligent environments, including smart cities, healthcare systems, and building management systems. This is because of recent improvements. It also shows how major IoT applications can affect the economy and the market share they are projected to control by 2025 [24]. Figure 3 shows the total number of connected devices with the IoT.

**Figure 4:**
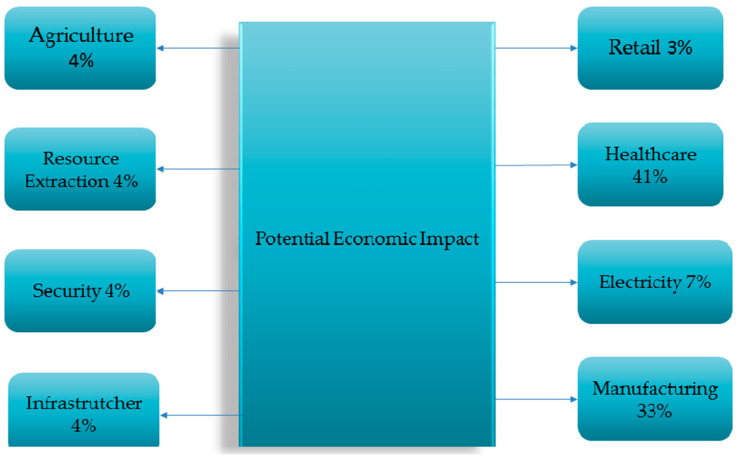
Potential economic impact of dominant IoT applications by 2025 [26].

The goal of these smart environments, which significantly impact business, society, and the economy, is to offer services based on IoT-enabled sensor data and clever methods. According to Navigant Research, the market for splitting city services will grow froM 93.5 billion USD in 2017 to 225.5 billion USD by 2026. Figure 4 shows the economic impact of IoT applications. The amount of available bandwidth, the number of users and smart objects in IoT networks, the ability to effectively manage large datasets, and the availability of scalable computing infrastructures, such as the cloud, are just a few of the factors that affect the quality of services offered by IoE applications in creative environments, such as intelligent cities [26].

As the digital world expands, both home appliances and industrial machines are becoming more intelligent. Security and privacy procedures that are effective in traditional networks could not be effective in the IoT. IoT connections’ versatility causes new security issues. We list a few examples below.

To enable the creation of reducing applications that could enhance people’s lives, the IoT aims to connect a sizable number of disparate devices. IoT devices come in various sizes and designs, requiring specific hardware and software solutions. The IoT connects billions of intelligent devices to real-world data in a way that has never been carried out before, regarding volume, speed, and organization [27].

The limitations of IoT devices and the dynamic and complex nature of the environment in which they operate exacerbate many of these concerns beyond the reach of standard security capabilities, even though many Internet access points share the majority of these problems [28] (as shown in Table 2).

A robust machine-learning-based IoT security system must consider the IoT cyber threat environment. Security features must therefore be designed and refined. A data-conditionality-reduction technique is essential because security features and the IoT data they are associated with have a direct impact on machine-learning-based security models [40]. “Feature engineering” refers to the process of developing and refining security features. This term is used when discussing the development of security models using machine learning. It may be challenging to accurately classify cyber dangers given the potential for irrelevant data in today’s IoT security datasets. If you use this kind of security model to make predictions, you could run into problems including high processing costs, excessive variation, the need to build the model, and a lack of generalization. Therefore, if an IoT security model with high-dimensional datasets comprise the right number of security features based on their impact or significance, it might be less stressful to design [39].

However, the Internet of Things raises security concerns because there are so many devices that can communicate to each other using different protocols. Internet of Things devices cannot be made safer because they do not have enough processing power. This means that the ways we currently protect IoT networks need to be greatly improved. In the last few years, security studies have paid a lot more attention to machine learning models. There may be a need for security for IoT systems because these devices regularly produce huge amounts of data that can be used to train machine learning algorithms [40]. New product components are developed using feature selection and principal component analysis, which together account for the majority of the significant data. These new brand elements could be useful for creating a machine-learning-based IoT security model [41]. Table 3 shows the dataset used for cybersecurity.

As the internet revolution continues, an increasing number of everyday objects and industrial tools begin to function as “smart” devices. Traditional data security and protection techniques are unlikely to work on IoT networks. The addition of new services to IoT networks introduces new security flaws. The goal of the IoT is to connect a wide network of various devices so that clamping software can be used to significantly improve people’s lives. IoT devices come in a variety of shapes and sizes, and they can perform a wide range of functions, necessitating the use of a wide range of hardware and software. A network of billions of connected computers makes up the IoT [58]. It also refers to the vast amount, rapid rate of change, and organization of data derived from the real world. The term “IoT” describes a network of devices capable of two-way data communication. As a result, any time and any place can be connected to an IoT network [59]. Theft of cookies, cross-site scripting, structured query language injection, session hijacking, and distributed denial of service attacks are all possible on connected IoT devices. DDoS assaults are especially dangerous for large, self-managed IoT networks [1]. IoT devices are temporary; thus, network configuration needs to be dynamic and flexible. Utilizing nearby devices, ad hoc networks can make communication over shorter distances easier. Proximity is described as how an IoT-enabled object responds and acts in relation to its actual surroundings [60]. Networks for industrial IoT encounter many difficulties. It is critical to have wireless connections that are speedy and reliable. Applications that call for low latency and high reliability connections include tracking, surgical equipment, and production on a production line [17]. An IoT device is a piece of hardware with a sensor that can send information to a remote location over the Internet. A complicated system must be built with the least amount of time, money, and effort possible because there are so many sensors involved in its operation [61]. Patient information is sensitive and valuable, making data security crucial in industries such as healthcare. Numerous IoT applications must make intelligent decisions in real time based on the preferences of the user [62].

Future-generation wireless networks must be reliable and self-sufficient. The individual’s use of technology in their daily lives is changing as a result of the IoT. Machine learning techniques are used by the Internet of Things to increase the effectiveness and independence of the network. Deep learning (DL) is a computationally costly and challenging machine learning (ML) technique. It is difficult to come up with strategies for combining deep learning technologies with IoT infrastructure to enhance the general performance of IoT applications. A range of methods that achieve a balance between computing costs and performance are needed for the next generation of IoT networks [63]. Machine learning techniques have quickly advanced, and they are presently used in a wide range of academic advancements [64]. For instance, they are carefully evaluated in a variety of sectors, including the cement business. Although cement enterprises in developing countries make a significant amount of money through the sale of valuable resources, they still face a number of difficulties. Optimization in machine learning has grown to be a significant topic of study in recent years. Using the FDH model, the set of production possibilities can be built in any way [65]. An innovative three-layer data-mining filtering pre-process for clustering techniques has been suggested by experts. It makes use of machine learning to increase accuracy and filter out irrelevant features and data. These stages of preparation were designed to reduce redundant information and improve precision. Finally, we are aware of the top business, best performance model and the most precise algorithm. The FDH model consistently performs at the highest possible degree of efficiency when compared to other suggested models [66]. Out of the three suggested filtering techniques, only the k-means algorithm consistently yields the best results. Second and third place, respectively, went to the model’s BCC and CCR. One of the most widespread technologies in modern society is the Internet of Things, which has a significant impact on people’s personal, professional, and financial lives. There is a lot of hope that the Internet of Things, both now and in the future, will enhance people’s lives in a variety of environments, from urban infrastructure to classrooms [67]. Automation, consumer comfort, and productivity have all risen as a result of these developments. Yet, threats and assaults have a big impact on the way intelligent Internet of Things applications perform. The quantity and complexity of threats to the Internet of Things have increased, and conventional approaches for protecting it have not been able to keep up [68]. The security system of the Internet of Things of the future must be dynamically updated so it is up to date for it to operate effectively. Artificial intelligence (AI), in particular machine learning and deep learning techniques, are required to make this viable. The author of [69] contrasted various approaches in order to identify the most effective one. We showed that this might be carried out interactively and how the model could be solved by switching the GDEA dual model to the MOLP. To solve the GDEA and identify the MPS within the bounds of each DMU’s efficiency, one may use this link as the foundation for an interactive MOLP technique. By fusing the STEM and DM methodologies, the GDEA dual model was able to demonstrate the preferences of the DM. In institutions for stroke care, the max-ordering method was applied to investigate the relationship between the GDEA dual model and the MOLP [67], which is a practical approach to securing IoT devices is machine learning. One of the most advanced AI techniques, machine learning, performs effectively in massively networked environments without explicit programming. The system may be trained to recognize and respond to various threats using machine learning techniques [13]. In this scenario, the majority of attacks might be stopped early on. Additionally, it appears that ML approaches may be useful for spotting new threats and putting strategic defenses in place. Machine learning algorithms may be employed in the future to create security standards for IoT devices, making them more dependable and user-friendly than they are now [25]. IDS’s effectiveness has led to a rise in popularity in recent years. Identification of people who do not belong in a particular location is the main purpose of an IDS [70]. Every host that tries to join the Internet of Things without authorization is considered an invader. IDS has not been studied enough. IDS on the IoT uses ML/DL in a variety of ways. Nonetheless, it struggles to deal with difficult problems. In addition, you can only apply these tactics for select types of blows, and they are not extremely accurate [40]. Right now, one of the biggest problems with the Internet of Things is that we do not fully comprehend how apps use data. This study introduces SAINT, a novel static taint analysis tool that locates weak data flows in IoT programmers. SAINT transforms the source code of an Internet of Things application into a lifecycle model. The access points, user inputs, events, and actions of the program are represented by this model. We then watch the information flow between sensitive inputs and final outputs in the washbasin while performing complete static analysis. Both the general SmartThings market and our specially created IOTBENCH application corpus were used to evaluate SAINT. In order to establish the value of SAINT and understand how the market normally functions, initial research focused on the SmartThings sector [71]. The second analysis used the IOTBENCH app corpus from the first one. Our analysis revealed that the great majority of currently accessible apps convey sensitive data, and that our system is capable of detecting taint sources and sinks. The outcomes of these tests also showed that our technology is able to identify the origin and final destination of contamination. This paper’s main focus is on architectural difficulties because they are the root cause of IoT’s poor performance and utility [72]. There are many problems and reasons to be worried. Communication, data management, zero-entropy systems, scalability, massive data collection, real-time data processing, security and privacy, interoperability, a lack of standardization, etc., are just a few of the problems that need to be solved. There were 20 billion connected things in 2014, and it was anticipated that this number would increase to 30 billion by 2020. These connections can be used in countless ways. The devices may have features in common, but they are made by different companies and run on different operating systems. Hadoop has trouble dealing with data sources that might carry out comparable operations but have wildly dissimilar data formats [71]. This lack of consistent standardization is summarized by the phrase “The Internet of Things May Never Speak a Single Language”. The lack of standardized protocols is now the greatest challenge in the path of the Internet of Things, according to a recent survey by Light Reading. This barrier needs to be removed because it prevents the growth of IoT interoperability. Technology progress, data standards, and wireless protocols have all been covered. Companies regularly create their own standards, which leads to incompatible technology [73]. One of the most important elements affecting people’s daily lives and well-being at work is “worker safety”. Studies that have been published in scholarly journals have shown that knowing that they are working in an environment where they are less likely to be in an accident improves employees’ emotions and well-being. It is crucial that all workplaces have proper safety precautions for their employees and operators, even though the industrial sector is the most dangerous for workers. No matter how frequent or unusual a job may be, it must always be protected in order to safeguard the workers’ health and safety. There are no published solutions that can also monitor and advise people during unusual or dangerous jobs, even if a range of technologies already meet these needs during “normal” operations (e.g., maintenance). The Internet of Things and other real-time applications and services, such as video surveillance systems, are growing quickly, showing the growing importance of technology in our daily lives. The Internet of Things and Industry 4.0 could help identify maintenance problems that have been noticed but not resolved. Fog devices are now processing a sizable percentage of IoT application processing thanks to the development of fog computing [74]. However, if fog nodes are underpowered, the device’s reliability may suffer and IoT apps will not be able to function. Many clear issues with read/write operations and unsafe edge settings must be addressed. Scalable fault-predictive proactive techniques are necessary to improve dependability. These algorithms should be capable of determining whether fog machines are not powered enough to work. The use of a recurrent neural network to predict proactive problems in fog devices when there are not enough resources is suggested in this research. The method makes use of a new rule-based network policy for computing, memory, and power, as well as an entirely theoretical long short-term memory. An LSTM network is used in the planned CRP to ascertain why the project failed due to a lack of finance. The proposed conceptual design also includes fault monitors and failure detectors. They guard against fog nodes failing to provide services to IoT applications. The accuracy of predictions on training data was 95.16 percent and on testing data, it was 98.69 percent when LSTM and the CRP network policy technique were coupled. Prior to this, machine learning and deep learning techniques were incomparable. This study uses vibration and acoustic emission sensor data to produce analyzable scalograms. To identify whether wavelet functions were useful, we used the RWE criterion. Further Sin GAN scalograms were produced, and a number of picture quality metrics were then retrieved and used to build feature vectors [75]. The experimental data required to train the LSTM model used to predict tool wear were insufficient. The feature vector was used to train the bidirectional, stacked, and vanilla LSTM models. We looked at five performance indicators, including root-mean-square error, mean square error, mean absolute error, and adjusted root-mean-square error to assess how effectively LSTM models can predict tool wear. The MAE, RMSE, and MSE were the lowest, with values of 0.005, 0.016, and 0.0002, respectively, despite the high values of R2 and Adj. It was discovered that the vibration signal’s R2 value was 0.997%. The findings show that the stacked LSTM model outperforms other LSTM models in predicting tool wear [76].

## 3. Methods and Materials

### 3.1. Research Method

The literature on IoT security studies has grown in recent years as more and more academics have developed an interest in the field. With the use of the AND OR search operators, we were able to find a vast amount of information that was relevant to topics, such as IoT, machine learning, deep learning, threats, cyberattacks, and vulnerabilities. We also included other terms, such as “blockchain”, “healthcare”, and “Data Mining. ML and DL”, in our search for a solution to the issue of IoT security breaches.

### 3.2. Exclusion and Inclusion

The IoT and machine learning approaches were used as a keyword string to find publications in databases from the IEEE, Springer, Scopus, Google Scholar, A.C.M., Science Direct, and Wiley. These works include research on machine learning categorization, IoT security, and the integration of health systems. Papers that were first chosen for review were peer-reviewed before being published. To better understand how machine learning works and how it might be used to improve IoT security, this research explored publications that concentrate on machine-learning-based approaches. After the initial search, any papers found were discarded. We only looked at a few articles because the review aimed to set standards for machine learning research criteria and methodology. The committee did not even read the additional recommendations.

#### Study Participants

**Figure 5:**
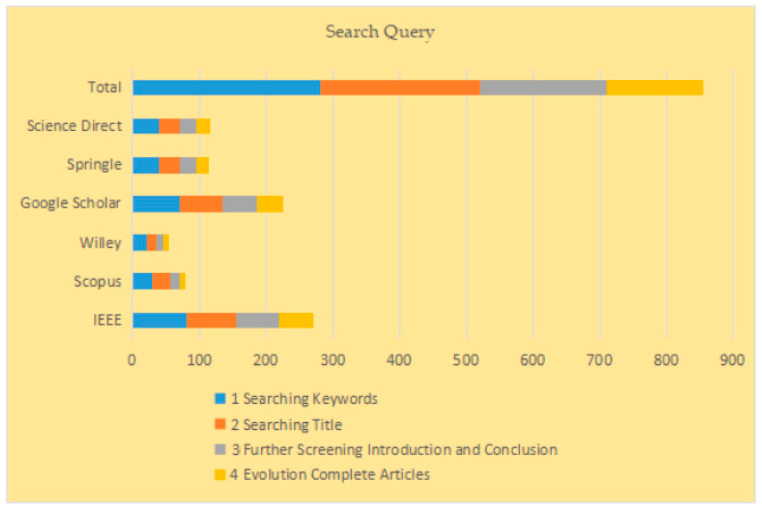
Search query.

The research query process is shown in Table 4 and Figure 5.

Table 5 shows the year-wise selection of papers.

**Figure 6:**
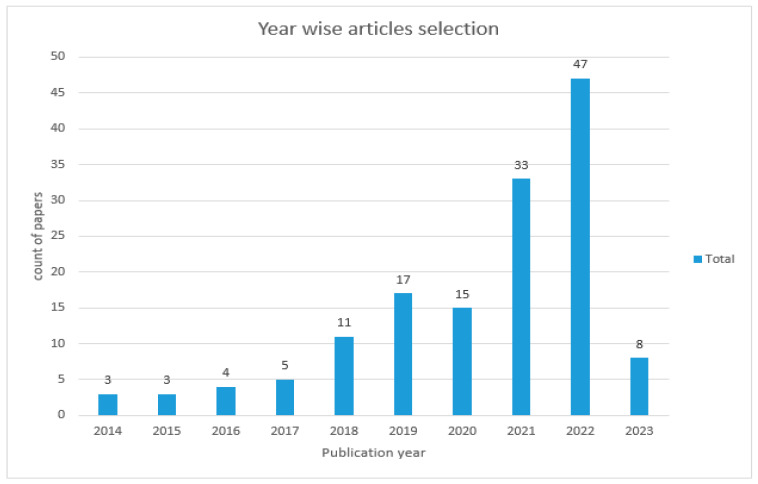
Year-wise article selection.

Figure 6 shows the year-wise article selection.

### 3.3. Research Questions

The research questions of the study are as follows:

1. What are the security issues of different IoT layers?
2. What are the deep learning methods used for IoT security?
3. What are the research issues and the future direction of IoT security?

### 3.4. IoT System Architectures and Security Concerns

#### 3.4.1. IoT Attacks on Surface Areas

We look at several possible attack paths for IoT systems and applications in the following sections. There are the following applications in particular: One of the most common entry methods for hackers is through IoT devices. Memory, firmware, physical interfaces, web interfaces, and network resources are only a few of the IoT systems’ many weak points. Hackers may obtain access through faulty parts, vulnerable update systems, and dangerous factory settings, to name a few. IoT devices may be attacked through the communication channels they use [77]. The protocols used by IoT systems may not be secure, which would put the plan in danger. IoT devices are vulnerable to network threats, including spoofing and denial of service. Security flaws in web applications and other IoT device software could provide unauthorized users access to the system. For instance, hackers might spread malicious firmware upgrades or steal user credentials using web applications [78].

#### 3.4.2. Architectures and Security Concerns

**Figure 7:**
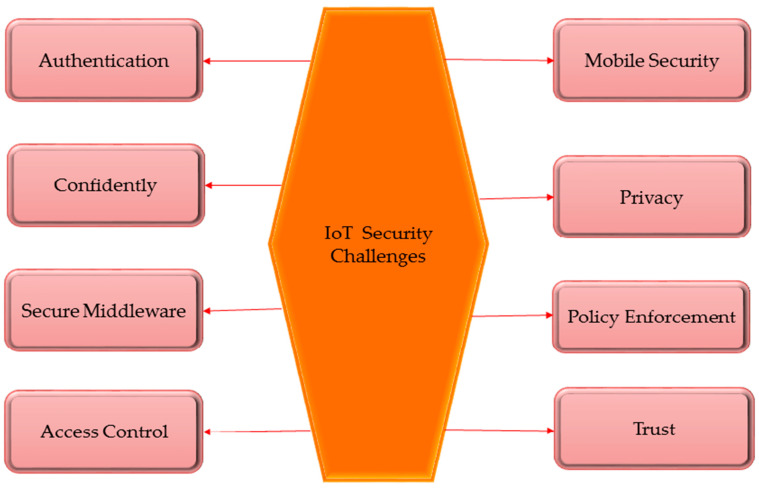
Security Challenges of IoT [81,82].

To highlight the security issues that affect the overall architecture of the IoT system, we summarize the IoT attack surface parts in this section. Different IoT concepts have been created by several academics and think tanks. A typical IoT design has three levels: perception, network, and application. However, it turns out that the support or middleware layer levels are vital because they must process data and draw wise conclusions [79]. A design for the IoT may contain a network layer and a support layer depending on its planned use. Many academic studies have also looked at how cloud computing might be used for the back-end architecture of the IoT [80]. Figure 7 shows the security challenges of IoT.

## 4. Results

### 4.1. Security Issues in the Perception or Sensing Layer

A conventional IoT design consists of three layers: the application layer, the network layer, and the perception layer [83]. However, the support or middleware layer between the network and application layers becomes more important as the significance of data processing and intelligent decision making rises. Multiple layers, including a network layer and a support layer, may be present in IoT systems. Cloud computing has been used as the underlying support layer in numerous studies of IoT systems.

Various sensors and other devices make up the perception layer, sometimes called the sensing layer. This layer’s storage, processing, memory, and communication capabilities are limited. The main methods this layer secures in the IoT network are node authentication, weak encryption, and access control [84]. Attacks and crimes against the perceiving layer’s privacy are too common in the real world. One approach to conduct this is to take control of a node. Malicious code usage, data injection, replay assaults, and side-channel attacks are other techniques. For example, if an attacker takes over a node, it will stop sending valid network data and may even stop using the IoT security program. It is possible that the IoT application will not operate as planned if it receives terrible data or is compromised by malicious code injection. A technique called eavesdropping, also called sniffing or snooping, allows an attacker to intercept and look through data being exchanged between two devices [85], as shown in Table 6. A replay attack in an IoT network could be defined as repeatedly falsifying, changing, or reusing the identities of related items. If an attacker has the required time and data encryption keys, they can execute a timing attack. There are a lot more ways than just direct node attacks for vital information to circulate [86].

#### 4.1.1. Issues with Networking and Data Communications Layer Security

The main goals of this layer are compatibility, privacy, and secrecy. At this layer, it is expected that criminal activities, including phishing, distributed denial-of-service attacks, attacks on data transit, routing attacks, identity authentication, and encryption, will occur [87]. This layer of the IoT is especially vulnerable to phishing attacks, which aim to obtain sensitive information such as passwords and login credentials. When an attacker or unauthorized user gains access to the IoT network while IoT apps gather and transfer sensitive data, this is characterized as an access attack, also known as a continuous advanced threat. Table 7 shows the attack and countermeasures on the data communication layer.

The most frequent and harmful kinds of network attacks are DoS and DDoS attacks. They use up network resources and compromise the operation of services. Malicious actors can also change routing channels’ routes when transmitting data by routing attacks, such as holes and worms [88].

#### 4.1.2. Security Issues in the Middleware or Support Layer

Distributed computing solutions have been used to replace centralized cloud environments in a variety of cases, with good results in terms of performance and response time. All sent data should now be checked for accuracy, concision, and secrecy.

When someone inside a network purposefully alters or steals data or information, this is known as a malicious inside attack [89]. By inserting malicious SQL queries into the code, SQL injection attacks are used to steal data from user services in the real world. When damage to one virtual machine spreads to another, this is a virtualization attack. With the help of cloud malware injection, a hacker can take over a cloud service, install malicious code, or even create a fake virtual machine. There could be significant consequences if attacks are so powerful that cloud infrastructure is incredibly frustrated [90]. Table 8 shows the attack and countermeasures on the support layer.

#### 4.1.3. Application Layer

Defining and maintaining IoT applications, including their interactions with specific clients, fall under the scope of the application layer. One way to use IoT services is through a user interface. A computer, a smartphone, or any other Internet-enabled smart device could serve as an interface. The data that the middleware layer process is used by the application layer [91]. This holds for a wide range of application categories, including applications for smart homes, smart cities, industry, construction, and health. The security needs of an application may change depending on how it functions. When sending information on climate change forecasts as opposed to when conducting online banking, it is acceptable to expect a better level of security. The application layer must address various security challenges, such as attacks on access control, malicious code, programming, data leaks, service interruptions, application vulnerabilities, and software flaws [92]. Table 9 shows the attack and countermeasures on the application layer.

Attacks that interrupt service, commonly referred to as “Distributed Denial of Service (DoS)” attacks, stop users from using IoT apps by sending a flood of requests to servers or networks. Threat actors could use sniffer software to monitor data being transmitted by IoT apps. Attacks that gain unauthorized access can seriously harm a system quickly by preventing users from using IoT-related services and wiping data [93].

**Figure 8:**
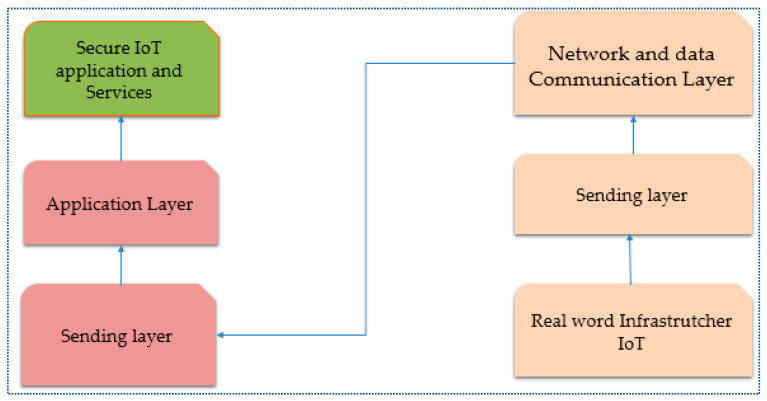
Layered Architecture [66].

Each layer of an IoT system may be vulnerable to different security flaws and attacks, as was already mentioned. Furthermore, there is a severe risk of unknown vulnerabilities. One must conduct a thorough investigation to find these hacks. Understanding artificial intelligence, especially machine learning and deep learning architectures and techniques, is an effective way to safeguard the system regarding IoT security. Figure 8 shows the layers and function of IoT architecture.

### 4.2. IoT Security Solutions Based on ML and DL

IoT devices can use AI technologies, such as machine learning and deep learning, to act correctly after learning from the data they gather. It is feasible to detect significant security event trends in IoT data using learning models, which frequently include rules, procedures, or complex “transfer functions” [94].

**Figure 9:**
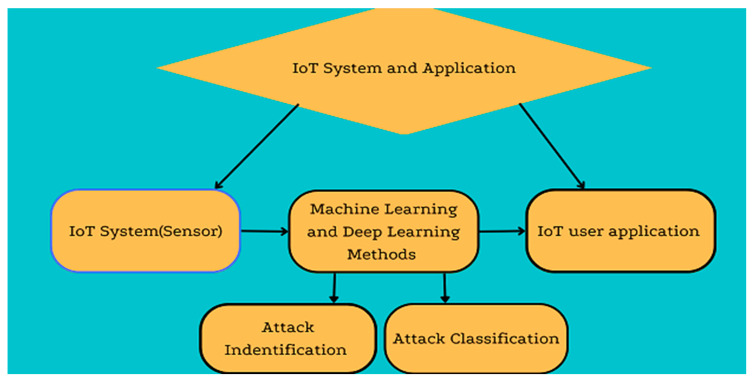
A machine learning security framework for IoT systems [96].

This enables DL and ML, which are entirely different, to function in real time over IoT networks. This shows how data-driven IoT security intelligence models could be created using ML and DL. IoT security data can be used to learn new things via classification and regression analysis, clustering, rule-based techniques, feature optimization, and DL with ANN, such as the M.N.L.P.N., C.N., and recurrent networks [95]. The following section covers the use of ML and DL to increase the security of IoT products. A machine-learning-based IoT security architecture is shown in Figure 9.

#### 4.2.1. Classification and Regression Techniques

Regression and classification techniques are well known and frequently used in IoT machine security. Predicting the outcome of discrete values or categories, such as anomaly, average, or attacks, is a standard definition of classification problems [97]. Regression is the technique of predicting a continuous or quantitative event, such as the effects of an attack. IoT security concerns include identifying intrusions and attacks, analyzing malware, and spotting fraud, as illustrated in Table 10.

The uses of such techniques are presented as follows:

- The SVM classification approach looks for unusual behavior in IoT devices and malware on Android to assure the dependability of IoT services [106].
- Anomalies, denial-of-service assaults, IoT intrusions, and irregularities in smart cities are all detected using the random forest approach [107].
- Two other methods for detecting abnormalities include a Naive-Bayes-based classification model and a linear-regression-based strategy for spotting malicious IoT malicious nodes [108].

Regression modeling, on the other hand, can be used to predict attacks or measure the severity of one. Worms, viruses, and another harmful software fall under this category [109]. Regression techniques, network packet characteristics, and quantitative security models that examine phishing over a specified period are examples of relevant models, as illustrated in Table 11.

iv. Any widely used R.T., such as linear, logistic, polynomial, and partial least-squares regression, can be used to build the quantitative security model. For instance, multiple regression analysis can create a correlation between human characteristics and how people desire to act in terms of cybersecurity [110].

#### 4.2.2. Clustering Techniques

Clustering is a standard method of unsupervised learning used in machine learning to analyze IoT security data. It may group or cluster data points based on similarity or dissimilarity metrics of security data from IoT devices from various sources. As a result, clustering might make finding hidden patterns and structures in data easier, making it simpler to spot anomalies or attacks in the IoT. Various perspectives, such as partitioning, hierarchies, fuzzy theory, distribution, and grids, can be used to cluster data. Many well-known methods for classifying data include k-means, K-medoids, and the Gaussian mixture model [111]. These clustering methods could be used to fix several IoT issues as illustrated in Table 12. An example of an algorithm used to profile unusual IoT device behavior is the k-means algorithm, which is one method that can be used to find outliers or noisy events is a dynamic threshold-based approach. Fuzzy clustering is frequently used to find IoT intrusions [112].

Cybersecurity applications can more effectively find helpful information or intelligence in system log data by clustering. Clustering algorithms may be very helpful in resolving IoT security issues, such as identifying outliers, anomalies, signatures, fraud, and cyberattacks, by exposing previously hidden patterns and structures in IoT security data [113].

#### 4.2.3. Rule-Based Techniques

Older patterns are less likely to stand out and aid in the identification or prediction of IoT security issues than newer unfriendly behavior patterns. Selectivity analysis, which examines current practices, may be more beneficial in some cases than conventional data analysis. Another critical goal is to develop a security model for IoT devices that is based on how recently they have been used. Innovative, portable IoT device solutions that take new data trends into account are required as part of our learning-based research on IoT security [114].

By creating various links and patterns based on support and confidence values, rule-based procedures are easy to use and complicate the model. The problem might be lessened with a robust association model. A rule-learning technique that can be used to find trustworthy, non-redundant links between ideas is shown in our earlier work [115]. Policy rules in a plan define which network usage is allowed and which is not. Even cyberattacks with no known vulnerabilities can be stopped by security policy monitoring filters and protections based on rules [116].

#### 4.2.4. Optimization of Security Features and Principal Component Analysis

In the current cyber threat environment, the development and optimization of security features are significant barriers to the success of an ML-based IoT security solution. Security characteristics and IoT data have a direct impact on ML-based security models, necessitating the use of a data-dimensionality-reduction technique. “Feature engineering” is the process of establishing and changing security features or variables so that machine-learning-based security models work properly. Today’s IoT security datasets may contain unused or irrelevant data, making simulation of cyberattacks and other challenges difficult [101]. The forecasting accuracy of a security model can be harmed by extreme variation, overfitting, expensive processing, and time-consuming model setup [93]. A high-dimensional dataset with many security attributes evaluated according to how important or relevant they are may make it easier to create an IoT security model [102]. Existing approaches include the correlation coefficient, the chi-squared test, and analysis of variance. Techniques for embedding information include regularization, Lasso, Ridge, Elastic Net, and tree-based feature importance [84]. Using feature selection and principal component analysis, it is possible to create new brand components that explain the most important data. As part of machine-learning-based security modeling, these enhanced signature properties may make it easier to manage large amounts of IoT security data, such as identifying anomalies in IoT network traffic [103].

#### 4.2.5. Multi-Layer Perceptron (MLP)

Deep learning usually uses the multi-layer MLP, FFAN. The input layer, the hidden output layers, and the actual output layer are the three layers that make up the traditional M.L.P. design. An AI network links each node in a layer to a specific value in the layer below it. In the end, this number is associated with the layer below it. As the model is being built, MLP employs backpropagation to adjust the internal weight values [117]. This M.L.P. network is used to analyze the NSL-KDD dataset’s malware, explain the IoT parameters, detect malicious traffic coming from IoT devices, and create a model for intrusion detection [118]. The idea divides network data into secure data and unsecure data.

#### 4.2.6. Recurrent Neural Network (RNN)

Another variety of artificial neural networks is the recurrent neural network. A directed graph representing time is constructed from the connections between the nodes. In the R.N.N. model, neural feed-forward networks are used. It looks at its internal state, or memory, to determine how long different input sequences last. IoT security, natural language processing, and speech recognition can all benefit from the RNN model’s capabilities to manage sequential data effectively [119]. IoT devices that are connected provide a lot of sequential data, including information that changes over time and network traffic flows. Recurrent connections in neural networks can uncover potential defense vulnerabilities when a threat’s communication patterns change over time. This is because it has a powerful model for predicting time series because of its long short-term Memory, which allows it to remember what it has been told in the past. For example, it is possible to identify and categorize dangerous applications and detect intrusions using an L.S.T.M.-model-based recurrent network [120]. It can also be used for further security-related tasks.

The detection and prevention of malware, spoofing, and computer virus attacks across a wide range of IoT devices can be made using a variety of deep learning models and hybrid network models [121]. One type of deep learning model that could be used to protect IoT devices is a DBN-based security model [122]. The authors looked at multiple approaches to in-depth learning. Additionally, they were referred to as unique features for jobs requiring human help and generative for those requiring none. Additionally, hybrid systems may be used if the data quality calls for it [123]. Data-driven security analytics in the context of the IoT can, therefore, greatly benefit from the above machine learning or deep learning methodologies, along with any lightweight modifications (as shown in Table 13)

### 4.3. Research Issues and Directions

As a result, through current and future research and development, we address the issues raised in this section and attempt to identify the best strategies for protecting IoT networks and devices. As a result, determining the best learning strategy for a specific IoT security scenario can be time consuming. This is conducted so that the results of various learning algorithms can differ depending on the quality of the input [84]. The model’s efficacy, precision, and labor requirements may be jeopardized if the incorrect learning method is used. Additionally, redundant IoT security data could lead to the gathering of irrelevant data and inaccurate conclusions. Machine learning or deep learning security models may not perform as well, be less accurate, or even be completely ineffective if the IoT data are incomplete in some way, such as by not being representative, being of poor quality, having irrelevant features, or being too small for training [134].

Here are a few possible future paths for study on IoT security:

Because of the way the IoT works, gathering security information can be difficult. A dynamic feature of the IoT known as heterogeneity was briefly discussed. It enables the routine collection of massive amounts of data from various sources. Data collection for IoT security is difficult. When working with IoT data, it is critical to understand the data collection process [62]. Statistics that are inaccurate or incomplete, outliers, and other flaws may jeopardize the security of the aging process or insufficient IoT devices [122]. The machine learning or deep learning methodology of IoT security has a significant impact on data quality and training availability, which has a significant impact on the IoT security model. IoT environments generate a lot of security data, which are hard to manage and clean up. Learning algorithms must be improved, or new data preparation techniques must be devised for them to be helpful in IoT security [135]. An effective IoT security solution must include the constraints or capabilities of IoT systems and devices. A device’s ability to store, compute, process, make decisions, and communicate must therefore be balanced with security. Therefore, choosing the best machine learning or deep learning algorithms requires extensive research [136]. In some cases, standard learning techniques might not work immediately with IoT devices due to the vast amount of repetitive processing. For example, the association rule learning approach may be used in a rule-based system to remove redundant IoT security data, making decision making challenging and ineffective [137].

#### 4.3.1. Poor Management

Systems based on the IoT are having trouble because of poor management. The problem is that most of the time, software engineers try to figure out how to extract useful data from sensors [138]. They do not care how data are gathered, just that it is. It is easier for attackers to hack a system and steal sensitive user data when there is no guarantee. Developers must start concentrating on data acquisition as a result [139].

#### 4.3.2. Naming and Identity Management

To communicate with other components of a network, each component needs to have its own identity. Therefore, a technique for dynamically identifying each network node with a special identification must exist [140]. When the IoT first started, IPv4 was used to give each networked device a special identifier. Because the number of Internet of Things devices is increasing, IPv6 is used to give each one a distinct name.

#### 4.3.3. Trust Management and Policy

The idea of trust is important and complicated. It is also necessary to have scalability, dependability, strength, and availability. It goes above taking safety procedures. IoT apps ask their users for sensitive information with their permission. Therefore, a privacy guarantee is necessary. User data are protected and cannot be accessed without permission. Academics have suggested a range of strategies for improving both trust and privacy in scholarly writings. These strategies for protecting trust and privacy in IoT applications have been ineffective. These issues are currently at the forefront of research on the Internet of Things as a result [141].

#### 4.3.4. Big Data

Currently, billions of devices are connected to the web, forming what is known as the IoT. Huge volumes of information are being generated by these devices. IoT struggles with the transmission and processing of massive datasets. Therefore, such a system is essential in order to solve the problem of big data [142].

#### 4.3.5. Security

Information security implementation in the IoT is challenging. Users communicate private data to complete tasks. There are various possible opponents for user privacy. Therefore, security measures should be implemented to safeguard user data and discourage unauthorized access [143].

#### 4.3.6. Storage

IoT devices must also be secure to use. Sensors keep an eye on the surroundings and send the information they gather to computers. Because there is no encounter measurement, the security of data storage devices cannot be guaranteed. As a result, there needs to be a way to stop unauthorized access to or monitoring of sensitive data [144].

#### 4.3.7. Authentication and Authorization

User IDs can be verified using several different techniques. The most common approach is to use a login and password, but there are other options as well, such as an access card, retina scan, voice recognition, or fingerprints. Authorization can also be obtained through access control. It is a method of protecting a system by only allowing those who need access to use it. The system has become complex because it consists of so many nodes and components. The traditional methods of authentication and permission have failed in large-scale networks. Although concerns with authentication and authorization have been researched, they still need to be fixed. To solve these challenges, such an approach is necessary [145].

#### 4.3.8. Secure Network

Man-in-the-middle and denial-of-service attacks are only two examples of the multiple ways the transport layer of a network can be used. An attack that prevents user’s access to the targeted system, device, or network resource is known as a denial-of-service attack [146]. A cyberattack known as “man-in-the-middle” occurs when an attacker pretends to be a third party and transmits and detects messages between two objectives who believe they are speaking directly to one another. Therefore, a set of protections must be put in place to guarantee the security of the network layer [147].

Therefore, it is challenging to create new, lightweight algorithms or procedures for IoT devices without first weighing the advantages and disadvantages of current teaching techniques [148].

Older patterns are less likely to stand out and aid in the identification or prediction of IoT security issues than newer unfriendly behavior patterns. Selectivity analysis, which examines current practices, may be more beneficial in some cases than conventional data analysis [136]. Another critical goal is to develop a security model for IoT devices that is based on how recently they have been used. Innovative, portable IoT device solutions that take new data trends into account are required. As part of our learning-based research on IoT security, we examined and evaluated the above study directions [149]. The security of the IoT can be improved by including context-aware computing; “context awareness” is a term used frequently in IoT computing to describe a system’s capacity to take in information about its surroundings and modify its behavior accordingly [150].

As a result, using chronological, geographical, individual, dependence, activity, the relationship between events or exchanges, and other contextual security data, it is possible to determine whether suspicious behavior occurs [151]. For example, a user may be able to connect to the network in the office but not when using public Wi-Fi. One area that could be investigated is how to create IoT security solutions that work in different contexts and adapt to them [152].

## 5. Conclusions

This research provides a comprehensive review of the literature on IoT security awareness. IoT model, IoT-based intelligent environments, and associated security challenges are some of the topics highlighted by machine learning solutions. In this work, we evaluated the knowledge base on IoT security intelligence. We investigated the IoT paradigm, IoT-based smart environments, security issues, and machine learning solutions to these problems. Identifying and protecting IoT devices and systems necessitates a thorough examination of IoT system architectures, as well as the cyberattacks that can break them down layer by layer. We investigated how various machine learning and deep learning technologies could be used to improve IoT security. If IoT security is to be effective, it must be built on machine learning or deep learning models that use data attributes. Before it can assist in making intelligent decisions, the system must have an effective learning algorithm based on the IoT security knowledge acquired and the application for which it is used. We also talked about potential directions and approaches for future research on teaching and learning. Because of these issues, there is room for new research in the field, and this is where the opportunity to develop effective strategies for continuously improving IoT security presents itself. We believe that our research on machine-learning- and deep-learning-based security solutions is a step in the right direction and will help other academics and practitioners find and implement IoT security solutions in the future.

## Figures and Tables

**Table 1:** List of abbreviations.

Abbreviations	Full Form
IoT	Internet of Things
MLP	Multi-layer perceptron
ML	Machine learning
DL	Deep learning
NB	Narrowband
LTE	Long-term evolution
DDoS	Distributed denial-of-service
DoS	Denial-of-service
ANN	Artificial neural network
KNN	K-nearest neighbors
RF	Random Forest
DT	Decision Tree
SVM	Support vector machine
NN	Neural Network
R.N.N.	Recurrent neural network

**Table 2:** IoT key issues.

References	IoT Key Issues	Advantages
[29]	Interoperability	General issues, IoT platforms and architectures, technical and semantic interoperability.
[30]	Security and privacy	Security and privacy issues, definition and design of secure IoT networks and architecture.
[31]	Management and control	IoT layer management and control, device, network, Application, data and trust management and control.
[32]	Architecture	Hardware, cloud centric, SOA, process architectures and conceptual models, application frameworks.
[29]	Quality of service (QoS)	Data traffic load, protocols for all layers in IoT architecture, QoS and QoE routine check.
[33]	Authentication and identification	Addressing issues and solutions, IoT integrations with internet protocols (IPv6), authentication, and identification issues.
[34]	Environment, power, and energy	Involvement of green technology in the IoT, design of low-power-consumption devices and chips, pollution control and management.
[35]	Smart city, healthcare, and transportation	Smart traffic management and control, smart devices for healthcare management, smart vehicles, energy management.
[36]	Data processing and storage	Data analysis, visualization, integration issues and solutions.
[37]	Reliability	Connectivity, mobility and routing issues, reliability of infrastructure and applications.
[38]	Scalability	Scaling issues on large platforms and geographical locations, potential discovery services.
[39]	Standardization	IoT definition, protocols design, architecture Standardization, vision and framework design.

**Table 3:** Datasets in the domain of cybersecurity.

Datasets	References	Datasets	References
NSL-KDD	[42]	Enron Spam	[43]
UNSW-NB15	[44]	Spam Assassin	[45]
DARPA	[46]	Ling Spam	[47]
C.A.I.D.A.	[48]	D.G.A.	[49]
ISOT’10	[50]	Malware Genome project	[51]
ISCX’12	[52]	Virus Share	[53]
CTU-13	[54]	Virus Total	[55]
C.I.C.I.D.S.	[56]	Comodo	[57]

**Table 4:** Research query process.

Phase	Process	Selection Criteria	IEEE	Scopus	Willey	Google Scholar	Sprinkle	Science Direct	Total
1	Searching	Keywords	80	30	20	70	40	40	280
2	Searching	Title	75	25	15	65	30	30	240
4	Further Screening	Introduction and Conclusion	65	15	10	50	25	25	190
5	Evolution	Complete Articles	60	10	5	40	20	20	155

**Table 5:** Year-wise selection of papers.

Publication Year	No of Papers
2014	03
2015	03
2016	04
2017	05
2018	11
2019	17
2020	15
2021	33
2022	47
2023	08

**Table 6:** Attacks and countermeasures on physical layer.

Layer	Types of Attacks	Description	Security Countermeasures
Physical	Eavesdropping	Infer information sent by IoT devices via network.	Faraday cage.
	Cyber-physical	Physically attacking a device.	Use of fault-detection algorithm to identify the faulty nodes.
	RFID Tracking	To disable tags, modify their contents, or imitate them.	Faraday cage.

**Table 7:** The attack and countermeasures on data communication layer.

Layer	Attacks	Description	Security Countermeasures
Data and Cloud services	Poisoning	Input of incorrect training data/labels to decrease the accuracy of classification/clustering process.	Data sanitization.
	Evasion	Generating an adversarial sample leading to evade system from detection spam and malware.	Retraining learning models by classifier designers with adversarial samples.
	Impersonate	Unauthorized access based on deep neural network DNN algorithm.	Defensive distillation on DNN.
	Inversion	Gathering information about ML models to compromise the data privacy.	Differential privacy (DP) technique and data encryption.

**Table 8:** The attack and countermeasures on support layer.

Layer	Types of Attacks	Description	Security Countermeasures
Transport	TCP flooding	Sending many packets through TCP protocol to stop or to reduce his activities.	A classifier based on SVM to detect and prevent DDoS TCP flooding attack.
	UDP flooding	Sending many packets through UDP protocol to stop or to reduce his activities.	A flow-based detection schema on router using a state machine and a hashing table.
	TCP SYN flooding	Tentative to open an externally connection without respecting to the TCP handshake procedure.	SYN-Cookies consist on coding client SYN message to change the state in the server side.
Network/ protocol	Man-in-the-middle	Violate the confidentiality and integrity in data transfer.	Intrusion-detection system (IDS) and virtual private network (VPN).
	DDoS	Making network resource unavailable for its intended use.	Ingress/Egress filtering, D-WARD, Hop Count Filtering and SYN-Cookies.
	Replay	Manipulate the message stream and reorder the data packets.	Timeliness of Message.

**Table 9:** The attack and countermeasures on the application layer.

Layer	Types of Attacks	Description	Security Countermeasures
Application	Malware	Gain access to IoT device by using a default Telnet or SSH account.	Disabling/changing default account of Telnet and SSH account.
	IRC Telnet	Forcing Telnet port to infect LINUX operating system of IoT device.	Disabling Telnet port number.
	Injection	Untrusted data are sent to an interpreter as part of a command or query.	Input validation control.

**Table 10:** Some ML techniques to handle various IoT security concerns.

Techniques	References
K-nearest neighbors	[98]
SVM	[99]
NB	[100]
AB	[101]
Logistic regression	[102]
D.T.	[103]
Intrude Tree	[104]
Behave D.T.	[105]

**Table 11:** Summary of Classification and Regression Methods.

	Algorithm	Complexity for Prediction	Advantages	Disadvantages	IoT Applications
Classification	KNN	O (np)	Easy to update in online Setting.	Unsalable to large data sets.	Smart Citizen, Smart Tourism.
	Naive Bayes	O (p)	Fast and highly scalable.	Strong feature independence assumptions.	Smart Agriculture, Spam filtering, text categorization.
	SVM	O (n sv p)	Good for unbalanced data.	The lack of transparency of results.	Real-time prediction: detection of intrusion, attacks, and malware.
Regression	Linear regression	O (p)	Processing under high rates	Very sensitive to outliers.	Energy applications, market prediction.
	SVR		Useful and flexible technique.	More complicated.	Intelligent transportation systems, Smart Weather.

**Table 12:** Summary of Clustering Techniques.

	Algorithm	Complexity	Advantages	Disadvantages	IoT Applications
Clustering	K-means	O (n2)	Very fast and highly scalable.	Difficult to predict the number of clusters (k-value).	Smart Cities, Smart Home, Smart Citizen, Intelligent Transport.
	DBSCAN	O (n2)	Fast and robust against outliers.	Performance is sensitive to the distance metric.	Smart Citizen, Smart Tourism.
	Feed Forward Neural Network	O (n2)	Non-linearity and robustness.	Longer time for training.	Smart Health.

**Table 13:** Summary of deep learning and machine learning algorithms [13].

Algorithm	Description
Naive Bayes	It is a collection of rules for grouping data into two or more categories. The term “naive” refers to the practice of calculating the probability of multiple hypothesis by making overly generalized claims. Because all the features are thought to be conditionally independent, determining their actual values is not necessary [124].
K-Nearest Neighbor	It is an efficient and straightforward technique for identifying connections between fresh and old data elements in a collection. After the model has been trained and classified, the degree of similarity between incoming input and its k neighbors is calculated [125].
K-Means Algorithm	The most used method is k-means clustering, which belongs to the unsupervised ML family. If the positive integer value of k is known, k-means clustering can sort or group devices according to them characteristics or parameters into k groups [126].
Random Forest and Decision Tree	It limits a model by placing restrictions on the properties of the data. Then, predictions for a further interesting independent variable are made using this model. Classification and regression issues can be addressed with a decision tree. These trees can be used to split datasets into several branches, each branch representing a rule [127].
Support Vector Machines	SVM is a technique to supervised machine learning that is simple to use and may be used for regression and classification. It can function in environments that really are binary and multi-class [128]. It divides the supplied data into groups using n dimensions and n + 1 hyperplane.
Recurrent Neural Networks	In order to address problems that cannot be resolved using conventional methods, this type of supervised learning involves the creation of a hierarchical network of decision-making components [129]. The programmer builds a network where a specified number of inputs lead to a predefined number of outputs. The multi-layer perceptron, convolutional neural network, and recurrent neural network are three types of neural networks that have been proposed [130].
Principal Component Analysis	Because it compresses data from several sources using an unsupervised manner, in huge datasets, it reduces the number of dimensions and extracts useful information as a set of “principal components” made up of unrelated variables. These components’ ranges are arranged from most variable to least variable, so the first component’s range contains the most variable data, and so forth. The parts that give the least data and variance can be removed to make things simpler [131].
Q-Learning	It is used to schedule spectrum management and IoT security resources. As well as for IoT security, a reinforcement learning method used in the field of machine learning is called Q-learning. In real life, an agent discovers the results of its acts through repeated attempts. It assesses the reward following each action and changes states appropriately [132]. There are rewards for good behavior and penalties for bad behavior.
Deep Learning	It functions as a feed-forward neural network in which there are no connections between any of the neurons in each layer. For deep learning, several layers are used, each having a higher level of abstraction than the layer before it [133]. One layer’s output is sent onto the next layer.

## Data Availability

Not applicable.

## References

[1] Hassija V., Chamola V., Saxena V., Jain D., Goyal P., Sikdar B. (2019). A Survey on IoT Security: Application Areas, Security Threats, and Solution Architectures. IEEE Access.

[2] Ślusarczyk B. (2018). Industry 4.0: Are we ready?. Pol. J. Manag. Stud..

[3] Sarker I.H., Kayes A.S.M., Badsha S., Alqahtani H., Watters P., Ng A. (2020). Cybersecurity data science: An overview from machine learning perspective. J. Big Data.

[4] Minerva R., Biru A., Rotondi D. (2015). Towards a definition of the Internet of Things (IoT). IEEE Internet Initiat..

[5] Khan M.A., Salah K. (2018). IoT security: Review, blockchain solutions, and open challenges. Future Gener. Comput. Syst..

[6] Minoli D., Occhiogrosso B. (2018). Blockchain mechanisms for IoT security. IOT.

[7] Zhang Z.-K., Cho M.C.Y., Wang C.-W., Hsu C.-W., Chen C.-K., Shieh S. IoT security: Ongoing challenges and research opportunities. Proceedings of the 2014 IEEE 7th International Conference on Service-Oriented Computing and Applications.

[8] Noor M.B.M., Hassan W.H. (2019). Current research on Internet of Things (IoT) security: A survey. Comput. Netw..

[9] Neshenko N., Bou-Harb E., Crichigno J., Kaddoum G., Ghani N. (2019). Demystifying IoT Security: An Exhaustive Survey on IoT Vulnerabilities and a First Empirical Look on Internet-Scale IoT Exploitations. IEEE Commun. Surv. Tutor..

[10] Abbas S.G., Hashmat F., Shah G.A. A multi-layer industrial-IoT attack taxonomy: Layers, dimensions, techniques and application. Proceedings of the 2020 IEEE 19th International Conference on Trust, Security and Privacy in Computing and Communications (TrustCom).

[11] Dalal K.R. Analysing the role of supervised and unsupervised machine learning in iot. Proceedings of the 2020 International Conference on Electronics and Sustainable Communication Systems (ICESC).

[12] Hussain F., Hussain R., Hassan S.A., Hossain E. (2020). Machine Learning in IoT Security: Current Solutions and Future Challenges. IEEE Commun. Surv. Tutor..

[13] Al-Garadi M.A., Mohamed A., Al-Ali A.K., Du X., Ali I., Guizani M. (2018). A Survey of Machine and Deep Learning Methods for Internet of Things (IoT) Security. IEEE Commun. Surv. Tutor..

[14] Zhou W., Jia Y., Peng A., Zhang Y., Liu P. (2018). The Effect of IoT New Features on Security and Privacy: New Threats, Existing Solutions, and Challenges Yet to Be Solved. IEEE Internet Things J..

[15] Li S., Xu L.D., Zhao S. (2015). The internet of things: A survey. Inf. Syst. Front..

[16] Asim M., Arif M., Rafiq M. (2022). Applications of Internet of Things in university libraries of Pakistan: An empirical investigation. J. Acad. Libr..

[17] Ma Z., Xiao M., Xiao Y., Pang Z., Poor H.V., Vucetic B. (2019). High-Reliability and Low-Latency Wireless Communication for Internet of Things: Challenges, Fundamentals, and Enabling Technologies. IEEE Internet Things J..

[18] Rawat D.B., Doku R., Garuba M. (2019). Cybersecurity in Big Data Era: From Securing Big Data to Data-Driven Security. IEEE Trans. Serv. Comput..

[19] Farrokhi A., Farahbakhsh R., Rezazadeh J., Minerva R. (2021). Application of Internet of Things and artificial intelligence for smart fitness: A survey. Comput. Netw..

[20] Yahya F., Zaki A.F.A., Moung E.G., Sallehudin H., Bakar N.A.A., Utomo R.G. (2021). An IoT-based Coastal Recreational Suitability System using Effective Messaging Protocol. Int. J. Adv. Comput. Sci. Appl..

[21] Routray S.K., Gopal D., Javali A., Sahoo A. Narrowband IoT (NBIoT) Assisted Smart Grids. Proceedings of the 2021 International Conference on Artificial Intelligence and Smart Systems (ICAIS).

[22] Sangra P., Rana B., Singh Y. (2023). Energy efficiency in IoT-based smart healthcare. Proceedings of Third International Conference on Computing, Communications, and Cyber-Security.

[23] Alshamrani M. (2021). IoT and artificial intelligence implementations for remote healthcare monitoring systems: A survey. J. King Saud Univ. Comput. Inf. Sci..

[24] Kumar Y., Singla R. (2022). Effectiveness of Machine and Deep Learning in IOT-Enabled Devices for Healthcare System. Intelligent Internet of Things for Healthcare and Industry.

[25] Tahsien S.M., Karimipour H., Spachos P. (2020). Machine learning based solutions for security of Internet of Things (IoT): A survey. J. Netw. Comput. Appl..

[26] Kaur J., Jaskaran, Sindhwani N., Anand R., Pandey D. (2023). Implementation of IoT in Various Domains, in IoT Based Smart Applications.

[27] Stracener C., Samelson Q., Mackie J., Ihaza M. (2019). The Internet of Things Grows Artificial Intelligence and Data Sciences. IT Prof..

[28] Chen L., Hu W., Jamieson K., Chen X., Fang D., Gummeson J. (2021). Pushing the Physical Limits of {IoT} Devices with Programmable Metasurfaces. 18th USENIX Symposium on Networked Systems Design and Implementation (NSDI 21).

[29] Noura M., Atiquzzaman M., Gaedke M. (2018). Interoperability in Internet of Things: Taxonomies and Open Challenges. Mob. Netw. Appl..

[30] Behrendt F. (2019). Cycling the Smart and Sustainable City: Analyzing EC Policy Documents on Internet of Things, Mobility and Transport, and Smart Cities. Sustainability.

[31] Kumar S., Tiwari P., Zymbler M. (2019). Internet of Things is a revolutionary approach for future technology enhancement: A review. J. Big Data.

[32] Li Y., Alqahtani A., Solaiman E., Perera C., Jayaraman P.P., Buyya R., Morgan G., Ranjan R. (2019). IoT-CANE: A unified knowledge management system for data-centric Internet of Things application systems. J. Parallel Distrib. Comput..

[33] Nawalagatti A. (2022). IoT: A Boon for Advancement of Technology. Int. J. Res. Appl. Sci. Eng. Technol..

[34] Ja S., Dhasb J.T.M., Angelc T.S. (2022). Proposed Novel Methodology for Automatic Drainage Block Identification in Smart Cities Using Internet of Things. Advances in Parallel Computing Algorithms, Tools and Paradigms.

[35] Muthulakshmi S., Chitra R. (2022). IoT technologies, applications and challenges, blockchain and its role in IoT: A survey. Int. J. Internet Technol. Secur. Trans..

[36] Singh P.K., Singh S., Usman H., Urooj S. (2022). Recent Advances and Future Trends of IoT-Based Devices. Energy Harvesting.

[37] Čolaković A., Salihović N., Dželihodžić A. (2022). Adaptive Traffic Management Systems Based on the Internet of Things (IoT). Proceedings of Advanced Technologies, Systems, and Applications VII: Proceedings of the International Symposium on Innovative and Interdisciplinary Applications of Advanced Technologies (IAT) 2022.

[38] Perwej Y., AbouGhaly M.A., Kerim B., Harb H.A.M. (2019). An Extended Review on Internet of Things (iot) and Its Promising Applications.

[39] Mazhar T., Irfan H.M., Haq I., Ullah I., Ashraf M., Al Shloul T., Ghadi Y.Y., Imran, Elkamchouchi D.H. (2023). Analysis of Challenges and Solutions of IoT in Smart Grids Using AI and Machine Learning Techniques: A Review. Electronics.

[40] Qureshi A., Qureshi M.A., Haider H.A., Khawaja R. A review on machine learning techniques for secure IoT networks. Proceedings of the 2020 IEEE 23rd international multitopic conference (INMIC).

[41] Mazhar T., Malik M.A., Haq I., Rozeela I., Ullah I., Khan M.A., Adhikari D., Ben Othman M.T., Hamam H. (2022). The Role of ML, AI and 5G Technology in Smart Energy and Smart Building Management. Electronics.

[42] Meena G., Choudhary R.R. A review paper on IDS classification using KDD 99 and NSL KDD dataset in WEKA. Proceedings of the 2017 International Conference on Computer, Communications and Electronics (Comptelix).

[43] Fernandes B.H.P. (2022). Smart Bed IoT-Based Wireless Data Acquisition for Untethered Patients. Ph.D. Thesis.

[44] Janarthanan T., Zargari S. Feature selection in UNSW-NB15 and KDDCUP’99 datasets. Proceedings of the 2017 IEEE 26th International Symposium on Industrial Electronics (ISIE).

[45] Karn R.R., Kudva P., Elfadel I.M. (2021). Learning Without Forgetting: A New Framework for Network Cyber Security Threat Detection. IEEE Access.

[46] Gharaibeh M., Papadopoulos C. (2014). Darpa-2009 Intrusion Detection Dataset Report. Tech. Rep..

[47] Ahmed M., Byreddy S., Nutakki A., Sikos L.F., Haskell-Dowland P. (2021). ECU-IoHT: A dataset for analyzing cyberattacks in Internet of Health Things. Ad. Hoc. Netw..

[48] Moustafa N. (2021). A new distributed architecture for evaluating AI-based security systems at the edge: Network TON_IoT datasets. Sustain. Cities Soc..

[49] Kilincer I.F., Ertam F., Sengur A. (2021). Machine learning methods for cyber security intrusion detection: Datasets and comparative study. Comput. Netw..

[50] Yavanoglu O., Aydos M. A review on cyber security datasets for machine learning algorithms. Proceedings of the 2017 IEEE International Conference on Big Data (Big Data).

[51] Amit I., Matherly J., Hewlett W., Xu Z., Meshi Y., Weinberger Y. (2018). Machine learning in cyber-security-problems, challenges and data sets. arXiv.

[52] Soheily-Khah S., Marteau P.-F., Bechet N. Intrusion Detection in Network Systems Through Hybrid Supervised and Unsupervised Machine Learning Process: A Case Study on the ISCX Dataset. Proceedings of the 2018 1st International Conference on Data Intelligence and Security (ICDIS), South Padre Island.

[53] Apruzzese G., Colajanni M., Ferretti L., Guido A., Marchetti M. On the effectiveness of machine and deep learning for cyber security. Proceedings of the 2018 10th international conference on cyber Conflict (CyCon).

[54] Putra M.A.R., Hostiadi D.P., Ahmad T. (2022). Botnet dataset with simultaneous attack activity. Data Brief.

[55] Folino G., Pisani F. (2016). Evolving meta-ensemble of classifiers for handling incomplete and unbalanced datasets in the cyber security domain. Appl. Soft Comput..

[56] Kasim Ö. (2022). A Robust DNS Flood Attack Detection with a Hybrid Deeper Learning Model. Comput. Electr. Eng..

[57] Kent A.D. (2015). Comprehensive, Multi-Source Cyber-Security Events Data Set.

[58] Xiao L., Wan X., Lu X., Zhang Y., Wu D. (2018). IoT security techniques based on machine learning: How do IoT devices use AI to enhance security?. IEEE Signal Process. Mag..

[59] Ngo Q.-D., Nguyen H.-T., Le V.-H., Nguyen D.-H. (2020). A survey of IoT malware and detection methods based on static features. ICT Express.

[60] Sarker I.H. (2021). Deep Cybersecurity: A Comprehensive Overview from Neural Network and Deep Learning Perspective. SN Comput. Sci..

[61] Kumar K.D., Sudhakara M., Poluru R.K. (2023). Towards the integration of blockchain and IoT for security challenges in IoT: A review. Res. Anthol. Converg. Blockchain Internet Things Secur..

[62] Kumar K.D., Venkata Rathnam T., Venkata Ramana R., Sudhakara M., Poluru R.K. (2019). Towards the Integration of Blockchain and IoT for Security Challenges in IoT. https://www.igi-global.com/chapter/towards-the-integration-of-blockchain-and-iot-for-security-challenges-in-iot/310448.

[63] Bin Zikria Y., Afzal M.K., Kim S.W., Marin A., Guizani M. (2020). Deep learning for intelligent IoT: Opportunities, challenges and solutions. Comput. Commun..

[64] Mirmozaffari M., Yazdani M., Boskabadi A., Dolatsara H.A., Kabirifar K., Golilarz N.A. (2020). A Novel Machine Learning Approach Combined with Optimization Models for Eco-Efficiency Evaluation. Appl. Sci..

[65] Mirmozaffari M., Shadkam E., Khalili S.M., Kabirifar K., Yazdani R., Gashteroodkhani T.A. (2021). A novel artificial intelligent approach: Comparison of machine learning tools and algorithms based on optimization DEA Malmquist productivity index for eco-efficiency evaluation. Int. J. Energy Sect. Manag..

[66] Sarker I.H., Khan A.I., Abushark Y.B., Alsolami F. (2022). Internet of Things (IoT) Security Intelligence: A Comprehensive Overview, Machine Learning Solutions and Research Directions. Mob. Netw. Appl..

[67] Mirmozaffari M., Shadkam E., Khalili S.M., Yazdani M. (2021). Developing a Novel Integrated Generalised Data Envelopment Analysis (DEA) to Evaluate Hospitals Providing Stroke Care Services. Bioengineering.

[68] Gupta C., Johri I., Srinivasan K., Hu Y.-C., Qaisar S.M., Huang K.-Y. (2022). A Systematic Review on Machine Learning and Deep Learning Models for Electronic Information Security in Mobile Networks. Sensors.

[69] Koroniotis N., Moustafa N., Slay J. (2022). A new Intelligent Satellite Deep Learning Network Forensic framework for smart satellite networks. Comput. Electr. Eng..

[70] Thomas L., Bhat S. (2021). Machine learning and deep learning techniques for IoT-based intrusion detection systems: A literature review. Int. J. Manag. Technol. Soc. Sci..

[71] Khan J., Khan J., Ali F., Ullah F., Bacha J., Lee S. (2022). Artificial intelligence and internet of things (AI-IoT) technologies in response to COVID-19 pandemic: A systematic review. IEEE Access.

[72] Ranaldi L., Pucci G. (2023). Knowing Knowledge: Epistemological Study of Knowledge in Transformers. Appl. Sci..

[73] Hasan N., Chen Z., Zhao C., Zhu Y., Liu C. IoT Botnet Detection framework from Network Behavior based on Extreme Learning Machine. Proceedings of the IEEE INFOCOM 2022-IEEE Conference on Computer Communications Workshops (INFOCOM WKSHPS).

[74] Montanaro T., Sergi I., Stefanizzi I., Landi L., Di Donato L., Patrono L. (2023). IoT-Aware Architecture to Guarantee Safety of Maintenance Operators in Industrial Plants. Appl. Syst. Innov..

[75] Haneef S., Venkataraman N. (2023). Proactive Fault Prediction of Fog Devices Using LSTM-CRP Conceptual Framework for IoT Applications. Sensors.

[76] Shah M., Vakharia V., Chaudhari R., Vora J., Pimenov D.Y., Giasin K. (2022). Tool wear prediction in face milling of stainless steel using singular generative adversarial network and LSTM deep learning models. Int. J. Adv. Manuf. Technol..

[77] Mendonça R.V., Silva J.C., Rosa R.L., Saadi M., Rodriguez D.Z., Farouk A. (2021). A lightweight intelligent intrusion detection system for industrial internet of things using deep learning algorithms. Expert Syst..

[78] Sadhu P.K., Yanambaka V.P., Abdelgawad A. (2022). Internet of Things: Security and Solutions Survey. Sensors.

[79] Tsimenidis S., Lagkas T., Rantos K. (2022). Deep learning in IoT intrusion detection. J. Netw. Syst. Manag..

[80] Alshohoumi F., Sarrab M., AlHamadani A., Al-Abri D. (2019). Systematic Review of Existing IoT Architectures Security and Privacy Issues and Concerns. Int. J. Adv. Comput. Sci. Appl..

[81] Jha K.K., Jha R., Jha A.K., Hassan M.A.M., Yadav S.K., Mahesh T. A Brief Comparison on Machine Learning Algorithms Based on Various Applications: A Comprehensive Survey. Proceedings of the 2021 IEEE International Conference on Computation System and Information Technology for Sustainable Solutions (CSITSS).

[82] Balo F., Torğul B. (2016). Internet of Things: A Survey. Int. J. Appl. Math. Electron. Comput..

[83] Albalawi A.M., Almaiah M.A. (2022). Assessing and reviewing of cyber-security threats, attacks, mitigation techniques in IoT environment. J. Theor. Appl. Inf. Technol..

[84] Deep S., Zheng X., Jolfaei A., Yu D., Ostovari P., Bashir A.K. (2020). A survey of security and privacy issues in the Internet of Things from the layered context. Trans. Emerg. Telecommun. Technol..

[85] Navya P., Rama G.S., Kumar T.P., Pasha S.N., Mahender K. (2022). IoT technology: Architecture, stack, security risks, privacy risks and its applications. Proceedings of AIP Conference Proceedings.

[86] Chatterjee U., Ray S. (2022). Security Issues on IoT Communication and Evolving Solutions. Soft Computing in Interdisciplinary Sciences.

[87] Haque A.K.M.B., Bhushan B., Dhiman G. (2021). Conceptualizing smart city applications: Requirements, architecture, security issues, and emerging trends. Expert Syst..

[88] Jangjou M., Sohrabi M.K. (2022). A Comprehensive Survey on Security Challenges in Different Network Layers in Cloud Computing. Arch. Comput. Methods Eng..

[89] Zahran S., Elkadi H., Helm W. (2022). Fog of Things Framework to Handle Data Streaming Heterogeneity on Internet of Things. Proceedings of International Conference on Advanced Intelligent Systems and Informatics.

[90] Rasheed M.A., Saleem J., Murtaza H., Tanweer H.A., Rasheed M.A., Ahmed M. (2022). A Survey on Fog computing in IoT. VFAST Trans. Softw. Eng..

[91] Yassein M.B., Shatnawi M.Q. Application layer protocols for the Internet of Things: A survey. Proceedings of the 2016 International Conference on Engineering & MIS (ICEMIS).

[92] Donta P.K., Srirama S.N., Amgoth T., Annavarapu C.S.R. (2021). Survey on recent advances in IoT application layer protocols and machine learning scope for research directions. Digit. Commun. Netw..

[93] Kakkar L., Gupta D., Saxena S., Tanwar S. (2021). IoT architectures and its security: A review. Proceedings of the Second International Conference on Information Management and Machine Intelligence.

[94] Ahmad Z., Khan A.S., Shiang C.W., Abdullah J., Ahmad F. (2020). Network intrusion detection system: A systematic study of machine learning and deep learning approaches. Trans. Emerg. Telecommun. Technol..

[95] Sarker I.H. (2021). Deep Learning: A Comprehensive Overview on Techniques, Taxonomy, Applications and Research Directions. SN Comput. Sci..

[96] Abbas G., Mehmood A., Carsten M., Epiphaniou G., Lloret J. (2022). Safety, Security and Privacy in Machine Learning Based Internet of Things. J. Sens. Actuator Netw..

[97] Han S., Mannan N., Stein D.C., Pattipati K.R., Bollas G.M. (2021). Classification and regression models of audio and vibration signals for machine state monitoring in precision machining systems. J. Manuf. Syst..

[98] Hsieh S.-C. (2021). Prediction of Compressive Strength of Concrete and Rock Using an Elementary Instance-Based Learning Algorithm. Adv. Civ. Eng..

[99] Aregbesola M.K., Griva I. (2022). A Fast Algorithm for Training Large Scale Support Vector Machines. J. Comput. Commun..

[100] Agghey A.Z., Mwinuka L.J., Pandhare S.M., Dida M.A., Ndibwile J.D. (2021). Detection of Username Enumeration Attack on SSH Protocol: Machine Learning Approach. Symmetry.

[101] Júnior E.C., Costa W.L., Portela A.L.C., Rocha L.S., Gomes R.L., Andrade R.M.C. (2022). Detecting Attacks and Locating Malicious Devices Using Unmanned Air Vehicles and Machine Learning. J. Internet Serv. Appl..

[102] Edemacu K., Kim J.W. (2021). Multi-Party Privacy-Preserving Logistic Regression with Poor Quality Data Filtering for IoT Contributors. Electronics.

[103] Puthal D., Wilson S., Nanda A., Liu M., Swain S., Sahoo B.P., Yelamarthi K., Pillai P., El-Sayed H., Prasad M. (2022). Decision tree based user-centric security solution for critical IoT infrastructure. Comput. Electr. Eng..

[104] Abdaljabar Z.H., Ucan O.N., Alheeti K.M.A. An intrusion detection system for IoT using KNN and decision-tree based classification. Proceedings of the 2021 International Conference of Modern Trends in Information and Communication Technology Industry (MTICTI).

[105] Al-Ghaili A.M., Kasim H., Al-Hada N.M. (2021). A secured data transform-and-transfer algorithm for energy internet-of-things applications. Telkomnika (Telecommun. Comput. Electron. Control).

[106] Menter Z., Tee W.Z., Dave R. (2021). Application of Machine Learning-Based Pattern Recognition in IoT Devices. Proceedings of International Conference on Communication and Computational Technologies.

[107] Meijin L., Zhiyang F., Junfeng W., Luyu C., Qi Z., Tao Y., Yinwei W., Jiaxuan G. (2021). A Systematic Overview of Android Malware Detection. Appl. Artif. Intell..

[108] Nakip M., Gelenbe E. (2022). Botnet attack detection with incremental online learning. Proceedings of International ISCIS Security Workshop.

[109] Ravikumar D. (2021). Towards Enhancement of Machine Learning Techniques Using CSE-CIC-IDS2018 Cybersecurity Dataset.

[110] Van der Schyff K., Flowerday S. (2021). Mediating effects of information security awareness. Comput. Secur..

[111] Sarker I.H. (2022). Machine Learning for Intelligent Data Analysis and Automation in Cybersecurity: Current and Future Prospects. Ann. Data Sci..

[112] AKTAR H., PERKGÖZ C. (2022). Autoencoder Aided Machine Learning Methods for Intrusion Detection Systems. New Trends in Technical, Natural Sciences, Engineering and Health Sciences.

[113] Giordano G., Palomba F., Ferrucci F. (2022). On the use of artificial intelligence to deal with privacy in IoT systems: A systematic literature review. J. Syst. Softw..

[114] Kallitsis M., Prajapati R., Honavar V., Wu D., Yen J. (2022). Detecting and Interpreting Changes in Scanning Behavior in Large Network Telescopes. IEEE Trans. Inf. Forensics Secur..

[115] Masum M.H.R. (2022). IT-Security Challenges for IoT Infrastructures.

[116] Gordaliza P.M. (2022). Computer-Aided Assessment of Tuberculosis with Radiological Imaging: From Rule-Based Methods to Deep Learning. Ph.D. Thesis.

[117] Elghamrawy S.M., Lotfy M.O., Elawady Y.H. (2022). An Intrusion Detection Model Based on Deep Learning and Multi-Layer Perceptron in the Internet of Things (IoT) Network. International Conference on Advanced Machine Learning Technologies and Applications.

[118] Uhricek D., Hynek K., Cejka T., Kolar D. (2022). BOTA: Explainable IoT Malware Detection in Large Networks.

[119] Madhu B., Chari M.V.G., Vankdothu R., Silivery A.K., Aerranagula V. (2022). Intrusion detection models for IOT networks via deep learning approaches. Meas. Sens..

[120] Al-Shareeda M.A., Manickam S., Saare M.A. (2023). DDoS attacks detection using machine learning and deep learning techniques: Analysis and comparison. Bull. Electr. Eng. Inform..

[121] Gopal S.B., Poongodi C., Nanthiya D., Kirubakaran T., Logeshwar D., Saravanan B.K. (2022). Autoencoder based Architecture for Mitigating Phishing URL attack in the Internet of Things (IoT) Using Deep Neural Networks. 2022 6th International Conference on Devices, Circuits and Systems (ICDCS).

[122] Bhattacharya S., Ghorai S., Khan A.K. (2023). Systematic Study of Detection Mechanism for Network Intrusion in Cloud, Fog, and Internet of Things Using Deep Learning. Human-Centric Smart Computing.

[123] Saheed Y.K., Baba U.A., Orje-Ishegh T., Longe O.B. (2022). An Efficient Machine Learning and Deep Belief Network Models for Wireless Intrusion Detection System. https://www.researchgate.net/publication/364203267_An_Efficient_Machine_Learning_and_Deep_Belief_Network_Models_for_Wireless_Intrusion_Detection_System.

[124] Deng L., Li D., Yao X., Wang H. (2021). Retraction Note to: Mobile network intrusion detection for IoT system based on transfer learning algorithm. Clust. Comput..

[125] Doshi R., Apthorpe N., Feamster N. Machine Learning DDoS Detection for Consumer Internet of Things Devices. Proceedings of the 2018 IEEE Security and Privacy Workshops (SPW).

[126] Zhang C., Sun X., Xia W., Zhang J., Zhu H., Wang X. (2022). Deep Learning Based Double-Contention Random Access for Massive Machine-Type Communications. IEEE Trans. Wirel. Commun..

[127] Anidu A., Obuzor Z. (2022). Evaluation of Machine Learning Algorithms on Internet of Things (IoT) Malware Opcodes. Handb. Big Data Anal. Forensics.

[128] Zhou W., Yu B. (2018). A cloud-assisted malware detection and suppression framework for wireless multimedia system in IoT based on dynamic differential game. China Commun..

[129] Chauhan J., Seneviratne S., Hu Y., Misra A., Seneviratne A., Lee Y. (2018). Breathing-Based Authentication on Resource-Constrained IoT Devices using Recurrent Neural Networks. Computer.

[130] Ismaeel H., Elmedany W. Anomaly-based detection Technique using Deep Learning for Internet of Things: A Survey. Proceedings of the 2022 International Conference on Innovation and Intelligence for Informatics, Computing, and Technologies (3ICT).

[131] An N., Duff A., Naik G., Faloutsos M., Weber S., Mancoridis S. Behavioral anomaly detection of malware on home routers. Proceedings of the 2017 12th International Conference on Malicious and Unwanted Software (MALWARE).

[132] da Costa K.A., Papa J.P., Lisboa C.O., Munoz R., de Albuquerque V.H.C. (2019). Internet of Things: A survey on machine learning-based intrusion detection approaches. Comput. Netw..

[133] Wahi V., Yadav S., Thenuia Y., Chauhan A. Anomaly Based Intrusion Detection for IoT. Proceedings of the 2022 3rd International Conference for Emerging Technology (INCET).

[134] Yang X., Peng G., Zhang D., Lv Y. (2022). An Enhanced Intrusion Detection System for IoT Networks Based on Deep Learning and Knowledge Graph. Secur. Commun. Netw..

[135] Thavamani S., Mahesh D., Sinthuja U., Manoharan G. (2022). Crucial attacks in internet of things via artificial intelligence techniques: The security survey. AIP Conference Proceedings.

[136] Khan R., Yang Q., Ullah I., Rehman A.U., Bin Tufail A., Noor A., Rehman A., Cengiz K. (2021). 3D convolutional neural networks based automatic modulation classification in the presence of channel noise. IET Commun..

[137] Haji S.H., Ameen S.Y. (2021). Attack and Anomaly Detection in IoT Networks using Machine Learning Techniques: A Review. Asian J. Res. Comput. Sci..

[138] Abideen Z.U., Mazhar T., Razzaq A., Haq I., Ullah I., Alasmary H., Mohamed H.G. (2023). Analysis of Enrollment Criteria in Secondary Schools Using Machine Learning and Data Mining Approach. Electronics.

[139] Khan W.U., Imtiaz N., Ullah I. (2020). Joint optimization of NOMA-enabled backscatter communications for beyond 5G IoT networks. Internet Technol. Lett..

[140] Esmalifalak M., Liu L., Nguyen N., Zheng R., Han Z. (2017). Detecting Stealthy False Data Injection Using Machine Learning in Smart Grid. IEEE Syst. J..

[141] Pourghebleh B., Wakil K., Navimipour N.J. (2019). A Comprehensive Study on the Trust Management Techniques in the Internet of Things. IEEE Internet Things J..

[142] Hajjaji Y., Boulila W., Farah I.R., Romdhani I., Hussain A. (2021). Big data and IoT-based applications in smart environments: A systematic review. Comput. Sci. Rev..

[143] Stergiou C., Psannis K.E., Gupta B.B., Ishibashi Y. (2018). Security, privacy & efficiency of sustainable Cloud Computing for Big Data & IoT. Sustain. Comput. Inform. Syst..

[144] Chui K.T., Liu R.W., Lytras M.D., Zhao M. (2019). Big data and IoT solution for patient behaviour monitoring. Behav. Inf. Technol..

[145] Mkrttchian V., Gamidullaeva L., Finogeev A., Chernyshenko S., Chernyshenko V., Amirov D., Potapova I. (2021). Big data and Internet of Things (IoT) technologies’ influence on higher education: Current state and future prospects. Int. J. Web-Based Learn. Teach. Technol. (IJWLTT).

[146] Khalil H., Rahman S.U., Ullah I., Khan I., Alghadhban A.J., Al-Adhaileh M.H., Ali G., ElAffendi M. (2022). A UAV-Swarm-Communication Model Using a Machine-Learning Approach for Search-and-Rescue Applications. Drones.

[147] Dehghantanha A., Choo K.-K.R. (2019). Handbook of Big Data and IoT Security.

[148] Dwivedi S.K., Roy P., Karda C., Agrawal S., Amin R. (2021). Blockchain-Based Internet of Things and Industrial IoT: A Comprehensive Survey. Secur. Commun. Netw..

[149] Yousafzai B.K., Khan S.A., Rahman T., Khan I., Ullah I., Rehman A.U., Baz M., Hamam H., Cheikhrouhou O. (2021). Student-Performulator: Student Academic Performance Using Hybrid Deep Neural Network. Sustainability.

[150] Raj A., Shetty S.D. (2021). IoT Eco-system, Layered Architectures, Security and Advancing Technologies: A Comprehensive Survey. Wirel. Pers. Commun..

[151] Gupta D., Juneja S., Nauman A., Hamid Y., Ullah I., Kim T., Tag eldin E.M., Ghamry N.A. (2022). Energy Saving Implementation in Hydraulic Press Using Industrial Internet of Things (IIoT). Electronics.

[152] Hussain A., Nazir S., Khan F., Nkenyereye L., Ullah A., Khan S., Verma S. (2021). Kavita A Resource-Efficient Hybrid Proxy Mobile IPv6 Extension for Next-Generation IoT Networks. IEEE Internet Things J..

